# Life skills evaluation in a kindergarten special education classroom

**DOI:** 10.1002/jaba.70041

**Published:** 2025-10-29

**Authors:** Jessica N. Torelli, Sara K. Snyder, Madeline L. Griffin, Madelynne P. Hellemn, Rachel R. Cagliani, Georgette A. Morgan

**Affiliations:** ^1^ Department of Communication Sciences and Special Education, Mary Frances Early College of Education University of Georgia Athens GA USA

**Keywords:** developmental disabilities, functional communication, life skills, prevention, social skills

## Abstract

Children with intellectual and developmental disabilities (IDD) are at risk of developing severe interfering behavior, such as aggression and self‐injury. Teaching young children with IDD life skills, such as social and communication skills, may help prevent the development of interfering behavior by addressing deficits in these areas. This study extended previous research on the Preschool Life Skills program by adapting it for young children with IDD, renamed the Life Skills Program (LSP). We evaluated the effects of researcher‐implemented LSP on the classwide use of social and communication skills and interfering behavior for five kindergarten children with IDD in a public special education classroom using a multiple‐baseline‐across‐units design. We also assessed generalization to novel settings and adults as well as maintenance of skills. The results demonstrated a functional relation between LSP and increased use of life skills along with preliminary evidence of skill generalization and maintenance over time.

Preschool Life Skills is a classwide preventive intervention program for typically developing young children in nonfamilial childcare settings who are at risk for developing interfering behavior (Fahmie & Luczynski, 2018; Hanley et al., 2007; National Institute of Child Health and Human Development, 2003). The program includes four units on instruction following, functional communication,^1^ tolerance, and friendship skills. These four units address 13 skills, selected in collaboration with kindergarten teachers, as skills essential for success in early elementary school and those commonly taught as functional alternatives to interfering behavior (Hanley et al., 2007). Studies evaluating Preschool Life Skills have sometimes used tiered instruction (e.g., Falligant & Pence, 2017; Robison et al., 2020). First, the skill is taught using whole‐group instruction and behavioral skills training (BST), followed by teaching trials with prompting, natural reinforcement, and descriptive praise (Tier 1). Children who do not master the skill with whole‐group instruction receive small‐group instruction (Tier 2). Small‐group instruction is formatted the same way as whole‐group instruction but includes fewer children in the instructional group. Children who do not master the skill after small‐group instruction receive one‐on–one instruction (Tier 3). One‐on–one instruction procedures have included tangible reinforcement (e.g., McKeown et al., 2021), token reinforcement (Kraus et al., 2012), or progressive intertrial intervals (Francisco & Hanley, 2012). Progressive intertrial intervals involve gradually increasing the duration of time between trials within a day to promote acquisition, generalization, and maintenance (Francisco & Hanley, 2012).

Researchers have begun extending Preschool Life Skills across cultures (e.g., Hálfdanardóttir et al., 2022) and to children with intellectual and developmental disabilities (IDD) who are at risk for developing persistent or severe interfering behavior (Simó‐Pinatella et al., 2019). For example, Falligant and Pence (2017) evaluated the level of instruction necessary for children with autism spectrum disorder (ASD) and developmental disabilities (4–6 years old) to acquire life skills using an abbreviated version of Preschool Life Skills and tiered instruction on five skills in a preschool. Two of the eight participants communicated using augmentative and alternative communication (AAC; Proloquo and sign language) and required Tier 3 instruction (one‐on‐one instruction) with modifications to meet mastery criteria for all five skills. However, participants who communicated using AAC were not the only ones who required modifications for successful skill acquisition. These findings provide preliminarily support that Preschool Life Skills can be modified for children with ASD and developmental disabilities who communicate using AAC.

Robison et al. (2020) extended the work of Falligant and Pence (2017) by teaching 12 skills from Preschool Life Skills, renamed Life Skills, to young children (3–9 years old) with IDD. They provided tiered instruction on life skills to nine participants, eight who communicated vocally and one who used an AAC device, in a private school for children with IDD. Use of life skills increased and errors decreased following tiered instruction. Gunning et al. (2020) extended Preschool Life Skills to parent delivery first for seven typically developing children and then for seven children (3–6 years old) with ASD. The researchers made modifications to the teaching context for children with ASD, including more frequent breaks, using activities with a discrete beginning and end, and systematic prompting. Results showed increases in skill use following instruction for typically developing children and those with ASD.

McKeown et al. (2021) focused specifically on friendship skills, evaluating the effects of different teaching approaches on friendship skill use for two typically developing children and two children with ASD (3–5 years old). They observed increases in friendship skills among typically developing children using BST with massed trials. However, both children with ASD required modifications to meet mastery criteria, which included adding progressive time delay prompting, an echoic controlling prompt, tangible reinforcement, and removing toys to eliminate potential competing reinforcement. This study also excluded children with interfering behavior and only included those who communicated vocally (i.e., echoing at least three‐word strings), engaged in consistent toy play, and followed instructions during play.

Collectively, studies evaluating life skills for children with IDD suggest that this population can learn life skills but may require modifications to the instructional approach to use the skills with fluency. They also point to several areas of need for additional research on Life Skills for children with IDD. First, only two studies (Gunning et al., 2020; Robison et al., 2020) implemented the entire Life Skills Program (LSP). The remaining studies evaluated a subset of skills from the program. It is possible that children with IDD require different types of instruction to master different kinds of skills and that teaching all skills provides synergistic benefits in preventing the development of interfering behavior. Additional studies evaluating the effects of the entire program for children with IDD are needed. Second, few participants across studies communicated using AAC, a common communication approach for children with IDD (American Speech‐Language‐Hearing Association, n.d.). Although there is a robust research base on teaching children with IDD to mand for objects using AAC, more research is needed to evaluate the effects of teaching children to use AAC for other types of social interactions (Logan et al., 2017). Third, across studies, all children with IDD required modifications to the initial teaching approach for at least one skill. These results suggest that group BST with massed practice trials may be insufficient for children with IDD to acquire some life skills. Furthermore, each study used slightly different instructional modifications to promote skill acquisition. Tiered instruction (Falligant & Pence, 2017; Robison et al., 2020) provides a method for organizing modifications and increasing the intensity of instruction only when needed. Evaluating a sequence of modifications that are likely to work for most children with IDD, arranged in a tiered approach, can promote the scaling up of LSP by informing a standardized adaptive protocol for implementation.

Finally, Preschool Life Skills was developed as a school‐based, interfering behavior prevention program. However, we are not aware of LSP studies for children with IDD that have evaluated the effects of the intervention at class‐ or schoolwide levels. Instead, several studies investigating the LSP, including those for children with IDD (e.g., Gunning et al., 2020; Robison et al., 2020), have used a multiple‐probe design in which individual child data are graphed as separate bars in a line graph format. This approach offers the benefit of showing individual variability in responding across units. However, the design of the graph limited visual analysis of within‐subject changes in level, trend, and variability over time. To promote our understanding of the intervention's effects and its potential for scale‐up, studies evaluating the effects of the LSP at the class‐ or schoolwide levels are needed. These studies may advance our understanding of the LSP for children with IDD and the extent to which instructional modifications may be necessary to promote skill acquisition. Classwide studies also provide an opportunity to study the extent to which these skills generalize and maintain, another understudied aspect of the LSP for children with IDD.

We sought to address these gaps in the literature on the LSP for young children with IDD. Specifically, we evaluated a LSP we designed for kindergarten students with IDD who communicated using AAC based on the program described in Robinson et al. (2020). First, we adapted, replaced, and added skills to increase the social validity and developmental appropriateness for early elementary children with IDD. Second, we refined the instructional methods by changing the prompting and error correction procedures. To extend the current body of research on LSP for children with IDD, we also investigated the generalization and maintenance of target skill use. Finally, we collected descriptive data on interfering behavior throughout the school day to gain preliminary information about the influence of LSP on interfering behavior (Fahmie & Luczynski, 2018). We conducted this evaluation of the LSP at the classwide level to better understand the effects of the LSP when implemented as a Tier 1 program.

Our primary research question was as follows: Is there a functional relation between the LSP and increased use of social and communication skills by young children with IDD, including those who communicate using AAC? We also addressed four secondary research questions: (1) Is there a functional relation between the LSP and decreased interfering behavior during probes for young children with IDD, including those who communicate using AAC? (2) Do social and communication skills maintain following completion of the LSP? (3) Do social and communication skill use and the absence of interfering behavior generalize to novel adults and settings? (4) Does interfering behavior throughout the school day decrease during and after the LSP?

## METHOD

### Participants

Table 1 describes participant characteristics. We conducted this study at the classwide level in a self‐contained kindergarten special education classroom. All five children in the class participated in the study. Each child's guardian provided informed permission to participate in the study. We followed our university's institutional review board policies for recruitment and obtaining consent. We monitored for dissenting behaviors throughout the study (e.g., refusal to transition to the play centers in which study activities were conducted) and waited for students to voluntarily walk to this area as a signal of assent. All participants received special education services under the educational eligibility of ASD and speech and language impairment. For all participants, the results of the Developmental Profile, 4th edition (DP‐4; Alpern, 2020) and Preschool Language Scales, 5th edition (PLS‐5; Zimmerman et al., 2011) yielded scores that fell within the delayed or severely delayed range. Scores on the Autism Spectrum Rating Scales (ASRS; Goldstein & Naglieri, 2009) or Autism Diagnostic Observation Schedule 2nd edition (ADOS‐2; Lord & Rutter, 2012) for all participants are reported in Table 1. All the previously mentioned assessment results were conducted by the school psychologist or speech language pathologist to determine special education eligibility.

**TABLE 1:** Participant characteristics.

Participant	Age	Race	PLS‐5	ASRS	ADOS‐2	DP‐4		VB‐MAPP		
						Cognitive (*SD*)	Adaptive (*SD*)	Milestones	Barriers	EESA
Kenneth	6	Black	50 (<.1)	‐	22	<50 (−3.33)	62 (−2.53)	50.50	47	15.50
Brendon	5	Black	50 (<.1)	72	‐	53 (−3.13)	84 (−1.07)	18.50	64	1
Roman	6	Multiracial	50 (<.1)	81	‐	<50 (−3.33)	65 (−2.33)	12.50	71	0
Simon	5	Multiracial	50 (<.1)	67	‐	55 (−3.00)	68 (−2.14)	80.50	39	67.50
Walter	5	Multiracial	50 (<.1)	67	‐	58 (−2.80)	71 (−1.93)	78	29	82.50

*Note*: PLS‐5 = Preschool Language Scales 5th Edition; ASRS = Autism Spectrum Rating Scale; ADOS‐2 = Autism Diagnostic Observation Schedule 2nd Edition; DP‐4 = Developmental Profile 4th Edition; VB‐MAPP = Verbal Behavior Milestones and Placement Program; EESA = Early echoic skills assessment.

Scores on the Verbal Behavior Milestones Assessment and Placement Program (VB‐MAPP; Sundberg, 2008) for Kenneth, Brendon, and Roman fell within the range of a Level 1 learner, suggesting that the focus of their intervention should be on mands, echoics, motor imitation, listener discrimination, tacts, play, spontaneous vocalizations, visual perception, and matching skills. The VB‐MAPP was completed by the student's classroom teacher. Simon and Walter's scores fell within the range of a Level 2 learner, indicating the skills of a Level 1 learner were present but still developing and suggesting the focus of intervention should be on systematically expanding those skills. Table 3 contains information regarding academic goals for each participant, topographies of interfering behavior, and their primary communication modalities. Academic goals for each participant were developed in collaboration with the student's special education team for their Individualized Education Program. The results of the VB‐MAPP and relevant academic standards as well as input from the students' caregivers and other related service providers guided goal development. All children communicated using the picture exchange communication system (PECS; Brendon, Roman, Simon, Walter; Frost, & Bondy, 2002), LAMP Words for Life (Prentke Romich Company, n.d.) software on an iPad (Kenneth), or one‐word vocalizations (Simon and Walter).

### Setting

The study took place in a kindergarten special education classroom in a Title 1 public elementary school in the southeastern United States. The classroom operated in partnership with the local university and was overseen by faculty and staff with doctoral‐level degrees in special education and Board‐Certified Behavior Analyst (BCBA‐D) certifications. The lead teacher held a master's degree in applied behavior analysis and BCBA certification. The classroom staff included four individuals pursuing master's degrees in applied behavior analysis. The staff member conducting teaching trials and probes with each child varied by session, as did the staff member leading instruction. The typical child‐to‐staff ratio in the classroom was one‐to‐one.

Whole‐group instruction took place at the front of the classroom at a table that was approximately 0.60 × 1.20 m and facing a wall‐mounted smartboard. The staff member leading instruction stood in front of the children, who all sat on the long side of the table, while the other classroom staff sat behind the children. On a typical day, five staff members were present in the classroom, with a total of 12 staff members rotating through the weekly schedule. All staff were trained on the experimental procedures before the study started and assisted in each tier of instruction and data collection throughout the study. The first and second authors conducted staff training using a BST format (Parsons et al., 2013), including a thorough description of the procedures, demonstration of the procedures, and role play of instruction and data collection with performance feedback. Although no formal criteria were established for each staff member, data collection and instruction were practiced until we observed no errors.

All teaching trials and probes during baseline, intervention, and maintenance phases took place in the play center of the classroom. The play center was approximately 1.50 × 1.50 m, with a variety of leisure items including cars, magnet tiles, Legos, action figures, etc. All leisure items were stored in plastic bins on shelves within the play center. Items used for teaching trials and probes varied by session. Staff used items following the child's lead (i.e., items they showed interest in or engaged with). Small‐group and one‐on‐one instruction sessions occurred at a kidney‐shaped table in the center of the classroom, about 0.60 × 1.80 m in size. The setting for generalization probes included the playground, elective classrooms (i.e., art, music, PE), and the cafeteria.

### Materials

We used paper‐and‐pencil forms for all data collection, including measuring dependent variables and procedural fidelity. To conduct teaching trials and probes, we used a variety of toys such as blocks, Legos, and action figures. We programmed different materials for generalization probes than for teaching trials and other probes, such as playground equipment and snacks (e.g., Goldfish, Cheerios). Occasionally, during generalization probes, a participant was highly engaged with an item that was included in teaching trials and probes. In these cases, we used the item during the generalization probe to capitalize on assumed motivating operations. Use of overlapping items between regular and generalization probes did not happen often but did happen occasionally for two participants (Simon, Roman) who showed interest in only a small range of items. Participants' AAC devices (i.e., iPad or PECS communication book) were within approximately 2 ft (0.61 m) of them during all teaching trials and probes across conditions.

### Life Skills Program overview

The LSP, adapted from Robison et al. (2020), included 13 life skills divided into four units. The units included instruction following, functional communication, tolerance of delays and denials, and friendship skills. Unit 1, Instruction Following, taught responding to name, following single‐step instructions, and following multistep instructions using a visual prompt. Unit 2, Functional Communication, taught requesting assistance, attention, items, and breaks. Unit 3, Tolerance Skills, taught tolerating (a) 30‐s delays, (b) 30‐s denials, and (c) the termination of preferred activities. Unit 4, Friendship Skills, taught saying thank you, greeting newcomers, and responding to sharing requests. We added two skills that were not included in Robison et al., requesting preferred items and requesting a break. The skills we adapted from Robison et al. were following multistep instructions (added visual cue), requesting attention (raise hand or say, “excuse me”), delays (no need to say, “okay”; limited to 30‐s delay), denials (no need to say “okay”; limited to 30‐s denials), termination of preferred activity (no need to say “okay”), and sharing (responds “yes” or “no” to sharing requests). We removed the skill of using a framed request following an appropriate attention recruitment. Table 2 provides operational definitions for each life skill.

**TABLE 2:** Life skills operational definitions.

#	Skill name	Description
**Instruction Following**		
1	Respond to name	Responds “Yes” within 2 s of name being called
2	Complete single‐step instruction^b^	Completes single‐step instructions within 10 s
3	Complete multistep instruction^b^	Completes two‐step instructions with visual cues within 10 s
**Functional Communication**		
4	Request assistance	Requests assistance with difficult task within 45 s by saying “Help me, please” vocally or using AAC
5	Request attention^b^	Requests attention by raising hand or saying “Excuse me” vocally or using AAC
6	Request an item^a^	Requests item by saying the item name vocally or using AAC
7	Request a break^a^	Requests a break by saying “Break” or “Stop” vocally or using AAC
**Tolerance of Delay and Denial**		
8	Tolerate delays^b^	Waits 30 s when delay is imposed without interfering behavior (may also say “Okay”)
9	Tolerate denials^b^	Waits 30 s when denial is imposed and alternative activity is presented without interfering behavior (may also say “Okay”)
10	Tolerate terminations^b^	Continues with classroom routine when preferred activity is terminated without interfering behavior (may also say “Okay”)
**Friendship**		
11	Say, “Thank you”	Says “Thank you” within 5 s of receiving an item vocally or using AAC
12	Acknowledge newcomers	Greets a newcomer within 10 s of their arrival by waving or saying “Hello”
13	Respond to sharing^b^	When someone asks to play or use an item the child is using, child either says “Yes” and gives the item, or says “No” vocally or using AAC

*Note*: AAC = alternative and augmented communication.

^a^ = skill added.

^b^ = skill adapted from the skills used in Robison et al. (2020).

### Response definitions and measurement systems

#### Probe data collection

Trained observers collected data using paper‐and‐pencil data sheets. For the Instruction Following, Functional Communication, and Friendship Units, they scored a trial as correct if the participant emitted the target skill independently (i.e., without prompting) within 10 s of initiating a trial (i.e., presenting a relevant evocative situation). For the Tolerance Unit, they scored a trial as correct if the participant engaged in the target response for the specified duration (e.g., waited without interfering behavior for at least 30 s). Trials were scored as incorrect if the participant (a) did not complete each component of the target skill; (b) emitted a behavior other than the target response; or (c) engaged in interfering behavior, even if this occurred with skill use. Interfering behavior included aggression, self‐injury, elopement, and screaming or crying, which were operationally defined for each participant. Aggression included behavior that was potentially harmful toward another such as biting, kicking, or hitting. Self‐injury included behavior that could cause injury to self, such as head banging on hard surfaces or head‐hitting. Elopement included leaving a teacher‐designated area without permission, and screaming or crying included vocalizations that were above a conversational level. In addition to scoring incorrect use of life skills (errors or engagement in interfering behavior), data collectors scored whether interfering behavior occurred during each trial (yes or no per trial).

#### Outside‐of‐session data

As a nonexperimental descriptive measure of generalization, observers also collected classwide data on the occurrence of interfering behavior throughout the school day using partial‐interval recording. Data collectors recorded the presence or absence of interfering behavior using pencil‐and‐paper sheets, divided into 10‐min intervals across the school day. They scored interfering behavior as present if any of the five participants engaged in interfering behavior during a given interval.

### Interobserver agreement

A second observer independently collected data during 65.79% of probe sessions with a minimum of 26% of sessions per participant and condition (baseline, intervention, maintenance) to assess interobserver agreement (IOA). We collected reliability during 5.70% of generalization probes. We assessed IOA for each behavior (correct responses, interfering behavior) using point‐by‐point agreement in which we scored whether observers marked the same response for each trial. We calculated IOA by summing the total number of agreements, dividing by the total number of opportunities, and multiplying by 100% to obtain a summary IOA percentage for the participant, unit, and session. We present IOA by behavior and by unit averaged across participants. Mean agreement for correct responses during baseline was 100%, and 99% (range: 87.5%–100%), 98.53% (range: 83.33%–100%), and 100% for Instruction Following, Functional Communication, Tolerance, and Friendship Units, respectively. Mean agreement for correct responses during intervention and maintenance was 99.56% (range: 86%–100%), 100%, 99.69% (range: 83%–100%), and 100% for Instruction Following, Functional Communication, Tolerance, and Friendship Units, respectively. Mean agreement for interfering behavior during baseline was 100%, 96.1% (range: 75%–100%), 96.21% (range: 33.33%–100%), and 100% for Instruction Following, Functional Communication, Tolerance, and Friendship Units, respectively. During intervention and maintenance, agreement for interfering behavior was 100% for all units except tolerance, which was 99.4% (range: 67%–100%). For generalization probes, mean agreement was 98.4% (range: 88%–100%) for correct responses and 100% for interfering behavior. We did not collect reliability data on observational partial‐interval recording of interfering behavior throughout the school day. It was not feasible for two classroom staff members to take this data across an entire day due to resource constraints.

### Experimental design and data analysis

We used a multiple‐baseline‐across‐units design at the classwide level to evaluate the effects of the LSP on participants' use of skills during probes. As a secondary purpose, we assessed the effects of the LSP on participants' interfering behavior during probes. Each probe data point represents the percentage of probe trials with correct responding for all skills in the unit. We conducted postunit probes after all children mastered all skills in the unit. In other words, we taught an entire unit to mastery before probing skills. We determined the order of instruction on units a priori, using the order reported in previous studies (i.e., instruction following, functional communication, tolerance of delay and denial, friendship skills; e.g., Hanley et al., 2014; Robison et al., 2020). We staggered the introduction of instruction across units using a response‐guided approach with a lag of at least three data points between each tier of the multiple‐baseline design. Our response‐guided approach involved observing for stability in the nonintervened tiers, along with an immediate increase in level with an increasing trend or stable and high‐accuracy responding in the intervened tiers before introducing intervention in the next tier. We visually analyzed data and inferred functional relations at the student and classwide levels. Student‐level analyses informed between‐participant variation in the response to intervention, whereas class‐level analyses informed the overall effect of the LSP on the group.

Because we did not conduct at least three generalization probes during baseline in some tiers, we descriptively analyzed these data using the same structured visual analysis procedure used for analyzing primary intervention effects but without inferring the presence or absence of functional relations and instead looking for effects suggestive of generalization or its absence.

### General procedures

**FIGURE 1:**
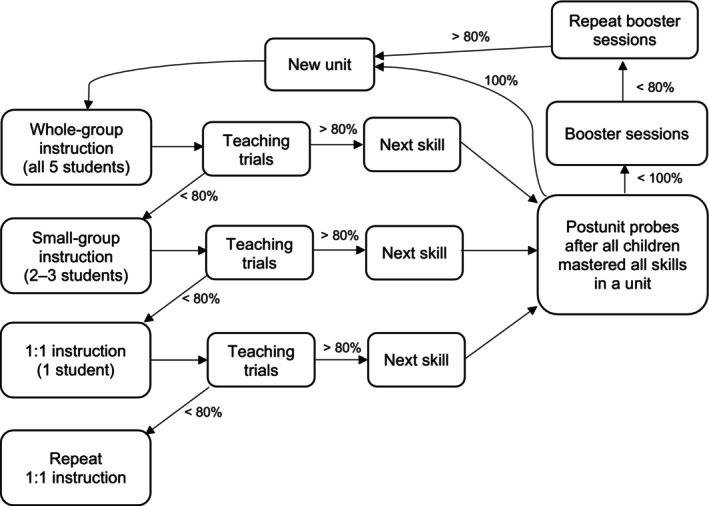
Life skills program instructional pattern.

Table 3 describes each of the 13 skills. Procedures were modeled after Robison et al. (2020). Instruction for each of the skills consisted of whole‐group instruction, small‐group instruction (if needed), and one‐on‐one instruction (if needed). Each instructional session was approximately 5 to 7 min in duration and was followed by a set of teaching trials discussed in greater detail below. Teaching trials occurred over a period of 10 to 30 min after instruction. We repeated this instructional model for each skill in a unit and then conducted postunit probes for all 13 skills. Figure 1 provides a flow chart of the instructional pattern. The mastery criterion for each skill was at least 80% correct responding for each participant during teaching trial sessions (eight of 10 trials correct for whole‐group and small‐group instruction, five of six trials correct for one‐on‐one instruction). If interfering behavior occurred during any trial, the staff member managed it in accordance with the child's classroom behavior plan, if applicable (Kenneth only), and waited until the child was calm for a minimum of 1–2 min before completing the next trial.

**TABLE 3:** Participant academic goals, interfering behavior topographies and communication modality.

Participant	Sex	Academic goals	Topographies of interfering behavior	Communication modality
Kenneth	Male	Identification of function of common objects, 1:1 correspondence, answering personal questions, answering reading comprehension questions	Aggression (pinching, biting, grabbing, kicking, hitting), self‐injury (head to surface)	LAMP on iPad
Brendon	Male	Receptive identification of letters, numbers, shapes, and colors	Aggression (scratching, biting, grabbing, pushing)	PECS Phase IV
Roman	Male	Matching colors, letters, numbers, and shapes	Aggression (scratching, hitting), elopement	PECS Phase IIIB
Simon	Male	Expressive identification of common objects, letter sounds, 1:1 correspondence, answering personal questions	Screaming or crying, elopement	PECS Phase IV and vocalizations
Walter	Male	Expressive identification of common objects, letter sounds, 1:1 correspondence, answering personal questions	Screaming or crying, elopement	PECS Phase IV and vocalizations

*Note*: LAMP = Language Acquisition through Motor Planning; PECS = Picture Exchange Communication System.

### Probe procedures (baseline, postunit, generalization, maintenance)

Procedures across all probe sessions for baseline, postunit, generalization, and maintenance were identical. During probe sessions, we assessed all 13 skills across the four units of the LSP. Staff members contrived evocative situations for all skills within the context of the play center in the classroom. Staff members did not provide any response prompts during probes. As in Robison et al. (2020), each skill was probed two or three times. If the child demonstrated the skill correctly or incorrectly in the first two trials, staff stopped presenting trials. If the child demonstrated the skill once correctly and once incorrectly, the staff member gave the child a third opportunity to demonstrate the skill. If the child engaged in the target skill, the staff member provided behavior‐specific praise and the natural consequence (e.g., saying “hello,” helping open a container, etc.).

We conducted postunit probes after all children mastered each skill within a unit. Following each postunit probe session, we conducted booster sessions if children made errors on a skill that they had already been taught. For example, during a postunit probe after Unit 2, if a child made an error on following single‐step instructions (a skill from Unit 1), we conducted a booster session for following single‐step instructions the next school day. If no errors occurred during a postunit probe session, no booster sessions occurred. Depending on the number of booster sessions children needed after a postunit probe, multiple school days sometimes elapsed in between probes sessions during which school staff conducted booster sessions. After completing booster sessions, we resumed conducting postunit probes until we obtained stable responding and introduced the next skill or moved to maintenance.

#### Generalization probes

Three university faculty members (first, fifth, and sixth authors) conducted generalization probes. Faculty members had no prior experience working with participants. They followed the same procedures for generalization probes as for all other probe sessions (e.g., all 13 skills across the four units were probed in each session without prompts). The settings for generalization probes varied across sessions and therapists, but sessions were mostly completed outside the classroom. The other settings included recess, elective classrooms (art, music, PE), and the cafeteria. We completed generalization probes throughout baseline (except Unit 1) and postunit probes, approximately every sixth session. Additionally, we conducted generalization probes every week we conducted maintenance probes.

#### Maintenance probes

We conducted maintenance probes after completing instruction on all units. At least 1 week passed between the end of instruction and the first maintenance probe, with the last maintenance probe occurring 3 weeks after the last day of instruction. We did not conduct booster sessions during the maintenance condition.

### Whole‐group instruction

Whole‐group instruction took place during morning circle, a whole‐group activity that was a part of the daily schedule for the kindergarten class. During whole‐group instruction sessions, a designated lead teacher stood in front of all children. The lead teacher first secured each learner's attention by delivering an attentional cue (e.g., “All eyes on me!”). Then, she began instruction modeled after BST including a short description of the skill, group practice of the skill, modeling the skill with another staff member, and role‐playing the skill with each child until they engaged in the target response. For skills that required a verbal response, the lead teacher modeled a vocal response and a visual model with picture cards on the child's personal picture exchange binder or corresponding button on their AAC device. The additional classroom staff who were sitting behind all children assisted the lead teacher in practicing the skill with each child. Staff provided error correction feedback using a least‐to‐most prompting (verbal, model, full physical) if the child did not perform the target skill correctly, consistent with prompting procedures used for other skills across the school day. They provided the natural consequence (e.g., delivered the item requested) and behavior‐specific praise to each child after they correctly engaged in the skill (e.g., “Great job looking when I called your name!”).

After whole‐group instruction sessions, children transitioned from the table area to the classroom play center. Supporting Information A provides examples of evocative situations used to practice life skills during play centers. Classroom staff contrived 10 evocative situations (teaching trials) corresponding to the skill staff taught in the whole‐group format. These trials did not begin until a minimum of 3 min had elapsed after whole‐group instruction. If the child engaged in the target response, the staff member provided behavior‐specific praise and moved on to the next trial with an intertrial interval of at least 30 s. If the child did not independently engage in the target response, as in instruction, the staff member repeated the trial with a verbal prompt, then a model prompt, and then a physical prompt if needed. For the class to move on to the next programmed skill, each child was required to independently engage in the target response, without any prompt, in at least 80% of trials. If a child did not meet mastery criteria they moved to small‐group instruction. Participants who met mastery criteria during the whole‐group instruction phase were in the play center or receiving other academic‐based instruction in a one‐on‐one format while the other students were receiving small‐group or one‐on‐one instruction.

### Small‐group instruction

Small‐group instruction included a smaller group of children than the initial whole‐group instruction. If all five children required further instruction (e.g., did not meet mastery criteria during whole‐group teaching trials), staff completed small‐group instruction with a group of three children and a group of two children. If just one child required more instruction, staff completed the small‐group instruction procedures with that single child. Thus, small‐group instruction ranged from one to three children in group size. The small‐group instruction procedures were identical to whole‐group instruction. There were at least two staff members present for small‐group sessions, one as the lead teacher and one available to model the skill or prompt children if necessary. Staff completed small‐group instruction at a small‐group table in which each staff member and a child sat around the table. After instruction, the children returned to the play center and staff completed 10 teaching trials as in whole‐group instruction. If the child still did not meet the mastery criterion during these teaching trials, they received one‐on‐one instruction.

### One‐on‐one instruction

We completed one‐on‐one instruction for two reasons: (a) the child did not meet mastery criteria after both whole‐group and small‐group instruction or (b) they failed a postunit probe for a particular skill (booster session). There were two staff members present for one‐on‐one instruction to model and prompt the skill. Staff conducted one‐on‐one instruction at the same table as small‐group instruction. After instruction, the staff member began a timer for 3 min and then began contriving teaching trials in the play center. Consistent with previous studies (Francisco & Hanley, 2012; Robison et al., 2020), we conducted six teaching trials following one‐on‐one instruction using progressive intertrial intervals. After the first trial, we completed each consecutive trial using the progression 10 s, 30 s, 2 min, 4 min, and 8 min. We repeated one‐on‐one instruction sessions until the child met the mastery criterion (>80% correct responding, or at least 5 of 6 trials correct).

### Procedural fidelity

We collected procedural fidelity data on probes, skill instruction (whole‐group, small‐group, one‐on‐one instruction), and teaching trials across units and participants. Across procedure type, an observer used a paper‐and‐pencil data form to mark whether each step of the programmed procedure was completed correctly or incorrectly. For probes, the procedures measured were (a) evocative situation setup, (b) prompt absence, (c) consequence delivery, and (d) trial number. For whole‐group and small‐group instruction, the procedures measured were (a) secure group attention, (b) skill description, (c) show each child the card or button on their communication device, (d) model, (e) role play with each child, (f) error correct if needed, and (g) descriptive praise for correct response. For one‐on‐one instruction, the procedures measured were (a) review target skill, (b) describe skill and point to card or button on child's device, (c) model, (d) role play, and (e) facilitate child engagement in highly preferred activity (or other appropriate evocative activity). For teaching trials, the procedures measured were (a) wait 3 min after the start of play center to initiate first trial, (b) present evocative situation, (c) present model prompt, (d) present physical prompt, (e) consequence delivery, and for on‐on‐one trials only, (f) intertrial interval length (steps c and d were required only for incorrect responses). We evaluated procedural fidelity for 100% of whole‐group, 89% of small‐group, and 41% of one‐on‐one instruction sessions. Mean fidelity was 100%, 97.88%, and 100% for whole‐group, small‐group, and one‐on‐one instruction sessions, respectively. We evaluated fidelity for 54% of teaching trials and 32% of probes with a minimum of 30% of sessions per unit and participant. For teaching trials, fidelity was 100%. For probes, mean fidelity was 99.75% (range: 60%–100%).

## RESULTS

**FIGURE 2:**
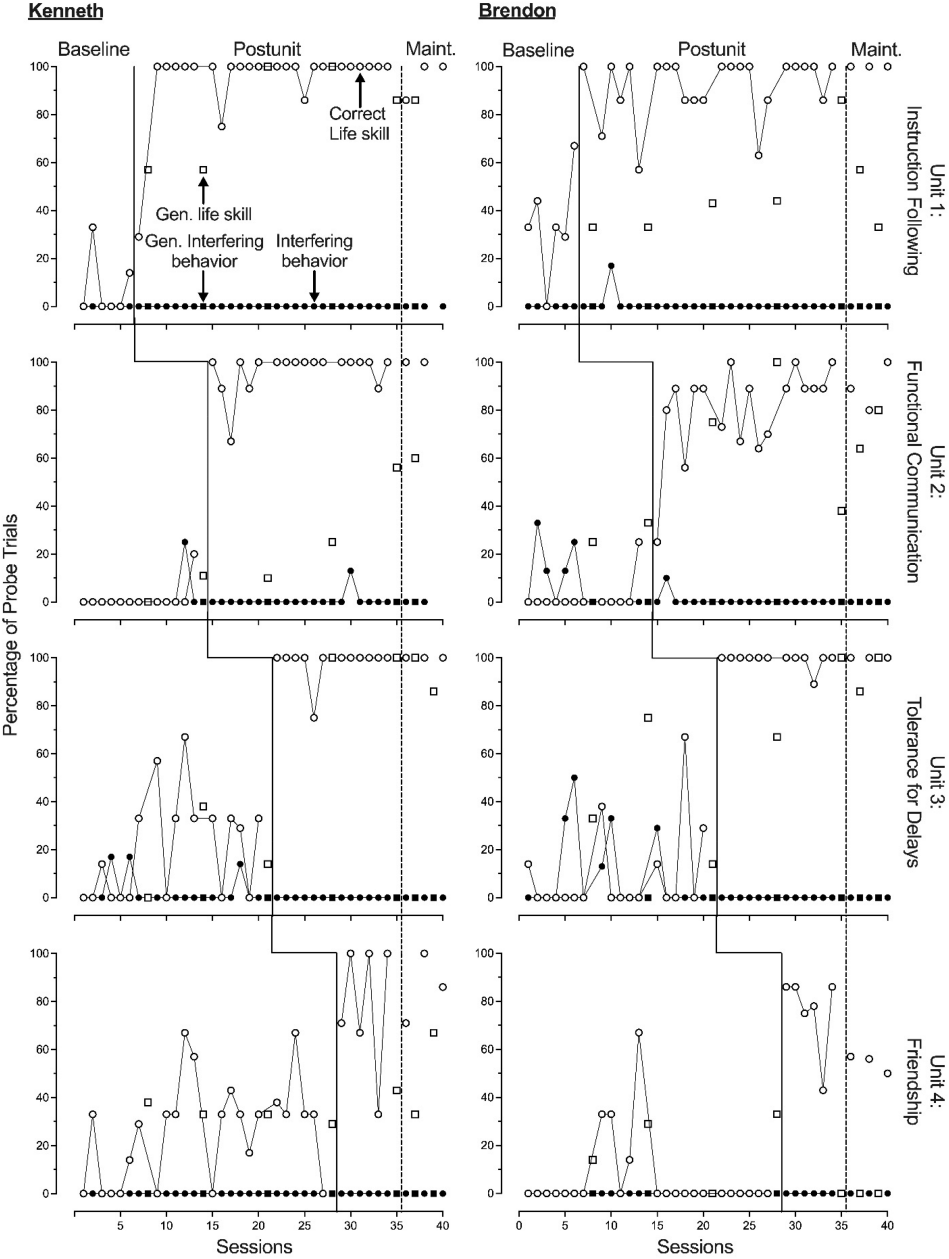
Kenneth and Brendon's treatment evaluation data. Gen. = generalization; Maint. = maintenance.

**FIGURE 3:**
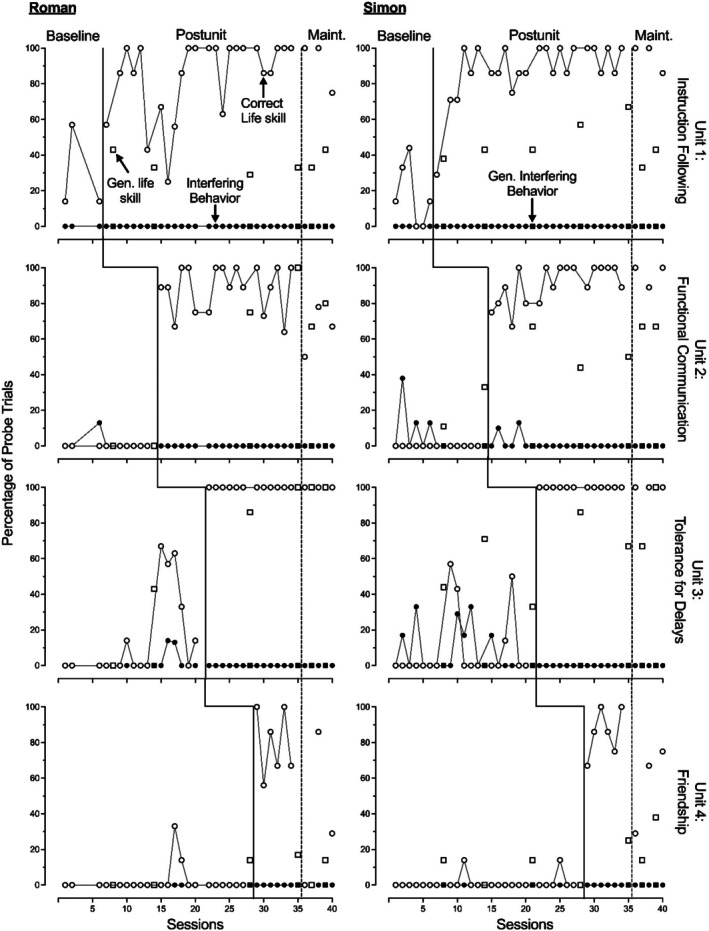
Roman and Simon's treatment evaluation data. Gen. = generalization; Maint. = maintenance.

**FIGURE 4:**
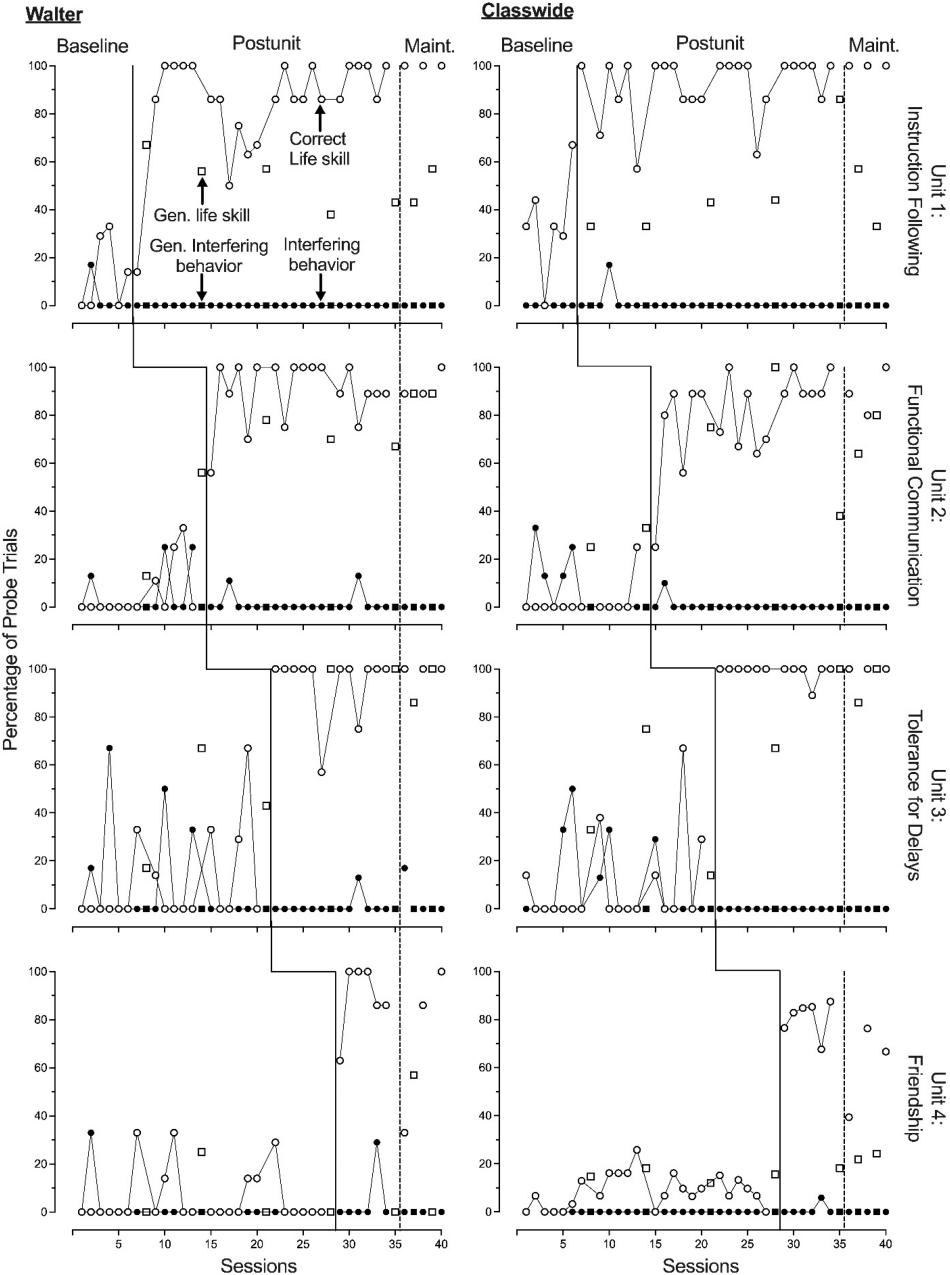
Walter and classwide treatment evaluation data. Gen. = generalization; Maint. = maintenance.

Figures 2, 3, 4 show the results of the LSP evaluation for correct life skill use (primary dependent variable) and interfering behavior (secondary dependent variable) during probes for each participant. For units containing three skills (Units 1, 3, and 4), each probe data point represents participant performance during two to three trials per skill, resulting in a total of six to nine trials. Similarly, for units containing four skills (Unit 2 only), each data point represents performance during eight to 12 trials across the four skills. Across all five participants, we observed low and stable or low and variable use of life skills during baseline, with an immediate increase in the correct use of life skills during postunit probes. We identified functional relations between units and increased use of life skills for all five participants.

Evidence of generalization and maintenance of skill use varied by participant. All five participants demonstrated maintenance for instruction following. We were unable to evaluate generalization for instruction following because we did not conduct these probes during baseline. All five participants demonstrated maintenance for functional communication and showed some evidence of generalization, with Kenneth and Simon showing lower levels of generalization. All five participants demonstrated maintenance for tolerance, and Kenneth, Brendon, Roman, and Walter demonstrated generalization. Simon showed limited generalization for tolerance. Only Walter showed strong maintenance of friendship skills, but Kenneth and Simon showed levels of maintenance slightly above baseline levels. Kenneth, Simon, and Walter showed some generalization of friendship skills. Roman and Brendon demonstrated low levels of correct responding during generalization probes of friendship skills across intervention and maintenance conditions. Overall, these results show that life skills often maintained across participants and provide preliminary evidence of generalization for some children and units.

For interfering behavior, we observed relatively low levels during baseline across participants. The primary exception to this was the Tolerance Unit, in which slightly elevated levels of interfering behaviors across participants, ranging from 0% to 50%, were observed. We also observed occasional trials with interfering behavior during baseline for Brendon, Simon, and Walter in the Functional Communication unit, resulting in variable baseline levels. During intervention, we consistently observed low levels of interfering behavior across units. Because interfering behavior was not consistently elevated across units for any participant during baseline, we could not infer the presence or absence of a functional relation between the LSP and decreased interfering behavior.

The right panel of Figure 4 shows classwide data. At the classwide level, we observed low levels of correct responding during baseline, with some variation across units. Baseline levels of correct skill use for Instruction Following, Tolerance, and Friendship units ranged from 0% to 40%, and skill use for functional communication remained below 10%. After introducing instruction, we observed an immediate increase in correct responding across all four units. Correct skill use ranged from 44% to 100% during intervention, with an increasing trend in two units (Instruction Following, Functional Communication), consistently high accuracy responding for Tolerance, and moderately high accuracy responding for Friendship skills. These results support the presence of a functional relation in which the LSP increased the correct use of target skills. Generalization probe data for correct responding during baseline were consistent with primary probe data. During intervention, correct responding during generalization probes increased from baseline but remained lower than other probes, except for Friendship skills, which remained at a similar level to baseline. These descriptive data suggest potential generalization of correct skill use for Instruction Following, Functional Communication, and Tolerance, albeit with lower levels of correct responding than during primary probes. Maintenance data suggest skill use maintained following the intervention.

**FIGURE 5:**
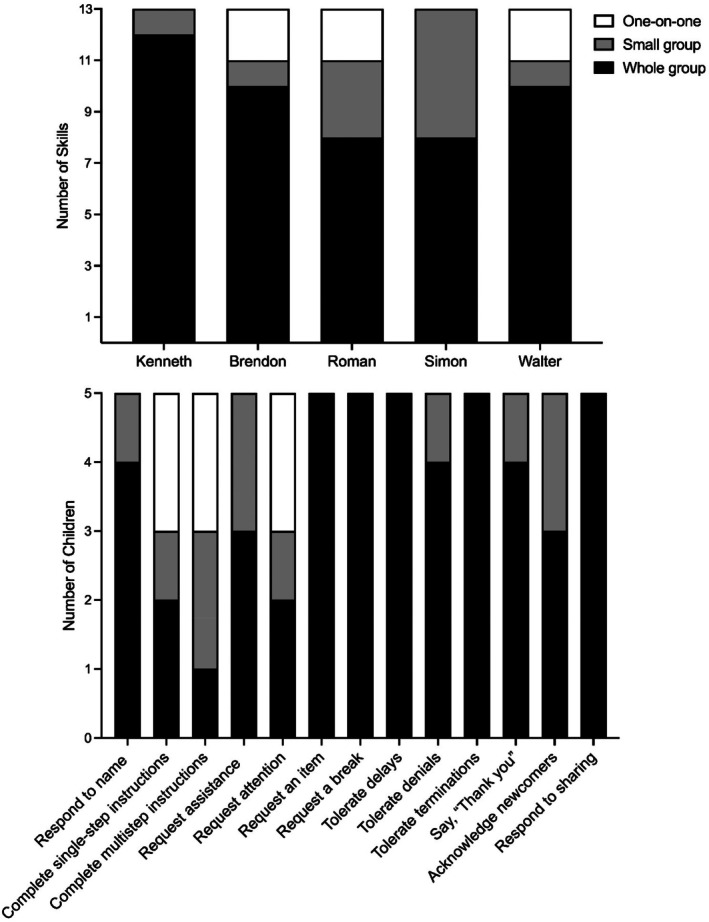
Participant skill acquisition by instructional tier.

All participants acquired the targeted skills with tiered instruction during the intervention, although the level of instruction required to acquire skills varied across participants. Most participants acquired most skills with whole‐group instruction, including evocative one‐on‐one teaching trials (*M* = 9.4 skills per child, range: 8–12). Participants acquired between 1–3 skills with small‐group instruction (*M* = 2 skills per child) and acquired the remaining 0–2 skills with one‐on‐one instruction (*M* = 1.2 skills per child). Figure 5 summarizes participants' skill acquisition by instructional tier. The top panel shows the number of skills each participant acquired by instructional tier, and the bottom panel shows the number of participants who acquired each skill at each instructional tier. Kenneth acquired all but one skill via whole‐group instruction, Brendon and Walter acquired 10 skills, and Roman and Simon acquired eight skills via whole‐group instruction. Brendon, Roman, and Walter required one‐on‐one instruction for two skills each, but Kenneth and Simon acquired all skills with whole‐group or small‐group instruction. By skill, the class acquired five skills with whole‐group instruction (requesting an item, requesting a break, tolerating delays, tolerating terminations, and responding to sharing) and five skills with whole‐group and small‐group instruction (responding to name, requesting assistance, tolerating denials, saying “thank you,” and acknowledging newcomers). Three skills required one‐on‐one instruction for participants to acquire them (completing single‐step instructions, completing multistep instructions, or requesting attention).

Despite frequent acquisition with whole‐group instruction, all participants required multiple booster sessions to maintain mastery over time. We conducted booster sessions for any skill in which participants exhibited an error during probes. Booster sessions occurred following and in between probe sessions, typically the school day after a probe session. Participants received 9–21 booster sessions across skills (*M* = 14 boosters per participant) and boosters for 6–11 skills (*M* = 8.2 skills per participant). These data suggest that participants required frequent practice with prompting to maintain acquisition of most skills.

Supporting Information B shows classwide data on interfering behavior collected throughout the school day using partial‐interval recording. We collected these data as an additional nonexperimental descriptive measure of the generalized effects of the LSP. These data are observational and may have been influenced by instructional procedures that occurred outside the context of the study. Interfering behavior occurred in 36%–75% of intervals during baseline and Instruction Following units (*M* = 61.6%). We observed a decrease in interfering behavior when we introduced the Functional Communication unit (*M* = 46.4%). This decreasing trend continued throughout the Functional Communication unit and maintained at a lower level (*M* = 27.4%) throughout the Tolerance and Friendship units.

## DISCUSSION

This study extended research on the LSP for young children with IDD, including AAC users, by evaluating the program's effects on social and communication skills and interfering behavior. The results demonstrated a functional relation in which the LSP increased the use of target skills for all participants and suggested skills generalized to novel adults and settings for all participants in at least some units. We observed low levels of interfering behavior during baseline and postunit probes across all units except the Tolerance unit in which interfering behavior decreased during postunit probes relative to baseline. Skills generally maintained following the completion of the LSP, although maintenance of friendship skills was limited. Data from generalization probes suggest that some generalization of life skill use may have occurred but to varying degrees across units. Descriptive data collected on interfering behavior across the school day show a decreasing trend in the percentage of intervals with interfering behavior across the course of the evaluation. As generalization data and interfering behavior data across the school day were both descriptive and solely observational, we cannot rule out alternative explanations for these findings. We tailored the LSP for this population by modifying the skills, teaching functional communication for common functions of interfering behavior, and simplifying friendship skills. We also adapted the prompting procedures from prior studies, adding least‐to‐most prompting.

Our results replicated and extended prior findings demonstrating that children with IDD vary in the level of tiered instruction needed to acquire life skills (Falligant & Pence, 2017; Robison et al., 2020). In our study, most children acquired most skills via whole‐group instruction and one‐on‐one evocative teaching trials. This is notable, as the treatment package was reasonably basic and unintrusive including BST, response prompting, error correction, natural reinforcement, and descriptive praise. We did not include supplemental reinforcers beyond natural consequences and descriptive praise. We hypothesize that using response prompting (least‐to‐most prompting) and implementing frequent booster sessions when participants exhibited errors contributed to the maintenance of life skills over time across units. Three participants required one‐on‐one instruction for completing single‐step instructions, completing multistep instructions, or requesting attention. We hypothesize one reason that completing single‐ and multistep instructions required modifications is that these skills occurred early in the LSP when participants had a limited learning history with the LSP teaching format. It is also possible that participants were learning other subskills involved in completing instructions, such as attending to attentional cues and verbal directives outside of the LSP, and needed more opportunities to practice these skills before they could complete single‐ and multistep instructions independently. For the skill of requesting attention, classroom staff reported that it was difficult to create an evocative condition for adult attention. Adult attention may not have functioned as a reinforcer for our participants, but children whose behavior is sensitive to adult attention may learn this skill without modifications. All participants mastered requesting items and breaks with whole‐group instruction, suggesting that they were able to acquire functional communication skills when the consequence was reinforcing. Importantly, we also observed that all children required booster sessions to maintain performance of numerous skills. These data suggest that frequent practice with prompting may be necessary to maintain consistent performance of most life skills for children with IDD.

In line with previous studies, we observed lower levels of correct responding in the Friendship unit relative to other units (Hanley et al., 2007, 2014). We based the friendship skills on Robison et al. (2020), adapting the final skill by teaching children to respond politely to sharing requests by saying, “yes” or “no,” rather than offering a toy to a newcomer within 10 s of arrival. Previous LSP studies teaching friendship skills have included more advanced skills, including acknowledging or complimenting others and comforting others in distress (Hanley et al., 2007, 2014). Despite teaching more basic friendship skills, participants still exhibited lower levels of correct responding on these skills. It is possible that friendship skills were more difficult than other life skills to learn for our participants. Other researchers have used supplemental reinforcers, such as tokens and stickers, to improve the acquisition of life skills (Beaulieu & Hanley, 2014; Beaulieu et al., 2012); this might be necessary for friendship skills in some circumstances. Other instructional modifications may also facilitate the acquisition of friendship skills, such as response restriction (e.g., placing only the appropriate picture card on the front of the child's PECS book) or using errorless teaching with progressive time‐delay prompts. Alternatively, it is possible the lower performance with friendship skills were due to fewer practice opportunities relative to other units. Several LSP studies have used a fixed sequence for introducing units in which friendship skills are introduced last. As a result, in the context of multiple‐baseline or multiple‐probe designs, there are fewer probes of friendship skills and, in the case of our study, fewer opportunities for booster sessions to practice skills.

Our results have several implications for future research and practice. First, combined with those of previous research (Falligant & Pence, 2017; Robison et al., 2020), the current results provide support for using tiered instruction to teach life skills to children with IDD, particularly when booster sessions are included. We recommend that practitioners use tiered instruction and embed regular practice with prompting to help children maintain skills. Second, our results point to the need to study efficient methods for maintaining mastery over time. Participants maintained high levels of correct responding during probes following booster sessions; however, booster sessions required one‐on‐one instruction and progressive intertrial intervals. This may not be realistic in many early elementary classrooms due to the intensive nature of booster sessions. These sessions took approximately 15 min per child and required two staff members to conduct. Future research may evaluate more resource‐efficient ways to maintain mastery and determine how much practice is necessary to maintain mastery (e.g., Wong et al., 2022). Third, our results align with those of previous studies that reported lower levels of correct responding for friendship skills. These results suggest that practitioners may need to use supplemental procedures and extra practice for children to master and maintain these skills. They also indicate a need for further research focused on the best approach for establishing friendship skills. Future researchers could consider exploring changes to the sequence of units and evaluating the effects of additional practice on performance. For example, in a single‐case design, researchers could counterbalance the sequence of skill units across classrooms, or in a group design, researchers could experimentally evaluate this question by randomly assigning different classrooms to different unit sequences (e.g., friendship skills first vs. last). Fourth, although our descriptive data on classwide interfering behavior throughout the school day show a decreasing trend, these data may have been influenced by other factors such as participants' continued exposure to classroom expectations and minimizing reinforcement for interfering behavior in the classroom. Future research may evaluate the effects of the LSP on interfering behavior by using group experimental designs, such as randomized controlled trials, to compare interfering behavior across the school day between LSP and control groups. These types of studies would also allow for evaluating the preventive effects of the LSP on interfering behavior over the long term. Finally, this study may be considered a preliminary evaluation of the LSP for children with IDD in early elementary school classrooms. We conducted this study in a public‐school classroom run in partnership with the local university. Thus, we had highly trained staff, including a lead teacher who was a doctoral student and BCBA; applied behavior analysis faculty oversight; and a 1:1 staff‐to‐student ratio of highly trained staff (i.e., applied behavior analysis master's students). Staff were able to implement the LSP with high levels of fidelity, which likely contributed to the positive outcomes. Future research should evaluate the effects of the LSP in public‐school classrooms with more typical resources and investigate the implementation supports needed to achieve similarly positive outcomes.

We found that LSP with tiered instruction increased the use of life skills for five kindergarten children with IDD who communicated via AAC. These skills maintained with frequent booster sessions including one‐on‐one instruction, naturalistic practice, and reinforcement using progressive intertrial intervals. We found evidence of generalization to novel adults and settings for all units except friendship skills. To enhance the effectiveness of the LSP in authentic school settings for children with IDD, future research may evaluate ways to improve acquisition of friendship skills and efficient ways to maintain skill use.

## AUTHOR CONTRIBUTIONS

The first author coordinated the project, conceptualized the experiment, led manuscript preparation and revision, and led data curation. The second author supervised data collector training and data collection and participated in project conceptualization, manuscript preparation, and manuscript editing. The third author supervised data collection and assisted with data curation. The fourth author collected data, curated the data, and assisted with manuscript preparation. The fifth and sixth authors collected data and participated in project conceptualization, reviewing, and editing the manuscript.

## CONFLICT OF INTEREST STATEMENT

The authors have no known conflicts of interest to disclose.

## ETHICS APPROVAL

This study was approved by the University of Georgia Institutional Review Board.

## Supporting information


**Data S1:** Supporting Information

## Data Availability

Data are available from the first author on reasonable request. Supporting Information A provides examples of evocative situations used to teach life skills, and Supporting Information B shows classwide data for interfering behavior throughout the school day.

## Footnotes

^1^
“Functional communication” is the naming convention for this program but is not representative of the extant literature for which a functional communication response is reinforced or taught to obtain the reinforcer identified via a functional analysis for interfering behavior.
