# Supplementary material for: Life skills evaluation in a kindergarten special education classroom
Source: J Appl Behav Anal. 2025 Oct 29;59(1):e70041. doi: 10.1002/jaba.70041 (PMC12689243; doi:10.1002/jaba.70041)

**Table S1**

*Example Evocative Situations for Training Life Skills*

| # | Skill name | Evocative situation |
| --- | --- | --- |
| Instruction following | |  |
| 1 | Respond to name | The student is engaged in play and a teacher calls their name. |
| 2 | Complete single-step instruction** | the teacher says, "put the block in the box." |
| 3 | Complete multi-step instruction** | The teacher says, "First put the block in the box, then close the box." |
| Functional communication | | |
| 4 | Request assistance | The student is given a toy or snack in clear, closed box that they cannot open independently |
| 5 | Request attention** | The teacher diverts their attention (e.g., engages with another student or turns away) |
| 6 | Request an item* | The teacher restricts access to a preferred item |
| 7 | Request a break* | The teacher provides instructional demands (e.g., gross motor, match to sample) |
| Tolerance of delay and denial | | |
| 8 | Tolerate delays** | The student requests an item, and the adult responds, “Wait, I’ll get it in a minute,” providing the item after a 30-s delay. |
| 9 | Tolerate denials** | The student requests to play with a toy a peer is engaged with, and the teacher responds, “Not right now, but you can do [alternate activity] instead,” while gesturing to the alternative activity. |
| 10 | Tolerate terminations** | A timer ends indicating the end of a preferred activity (e.g., play centers, recess, specials, snack), and the teacher says, "All done with [preferred activity], time to do [work activity name]." |
| Friendship | | |
| 11 | Say, "Thank you" | A teacher or peer gives the child a preferred item, snack, or assistance (e.g., handing over a toy or helping open a package). |
| 12 | Acknowledge newcomers | A peer or teacher walks over and joins an activity the student is already engaged in. |
| 13 | Respond to sharing** | A peer or teacher approaches and asks, “Can I play with that?” while the student is actively using a toy. |

*Note.* *Indicates skill added, **indicates skill adapted from the skills used in Robison et al. (2020)

**Figure S1**


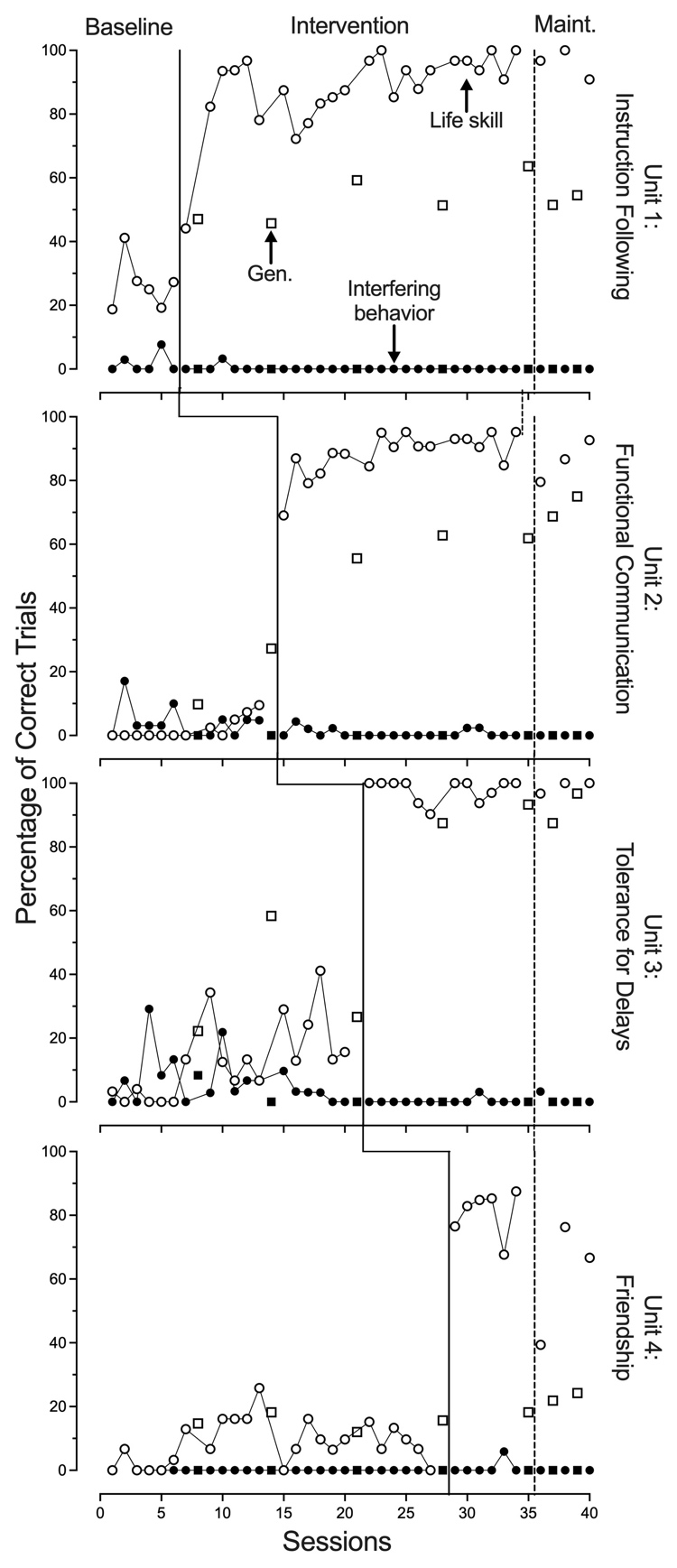
*Class-Wide Treatment Evaluation Data*

*Note*. Gen. = generalization; maint. = maintenance

**Figure S2**

*Class-Wide Interfering Behavior Throughout the School Day*


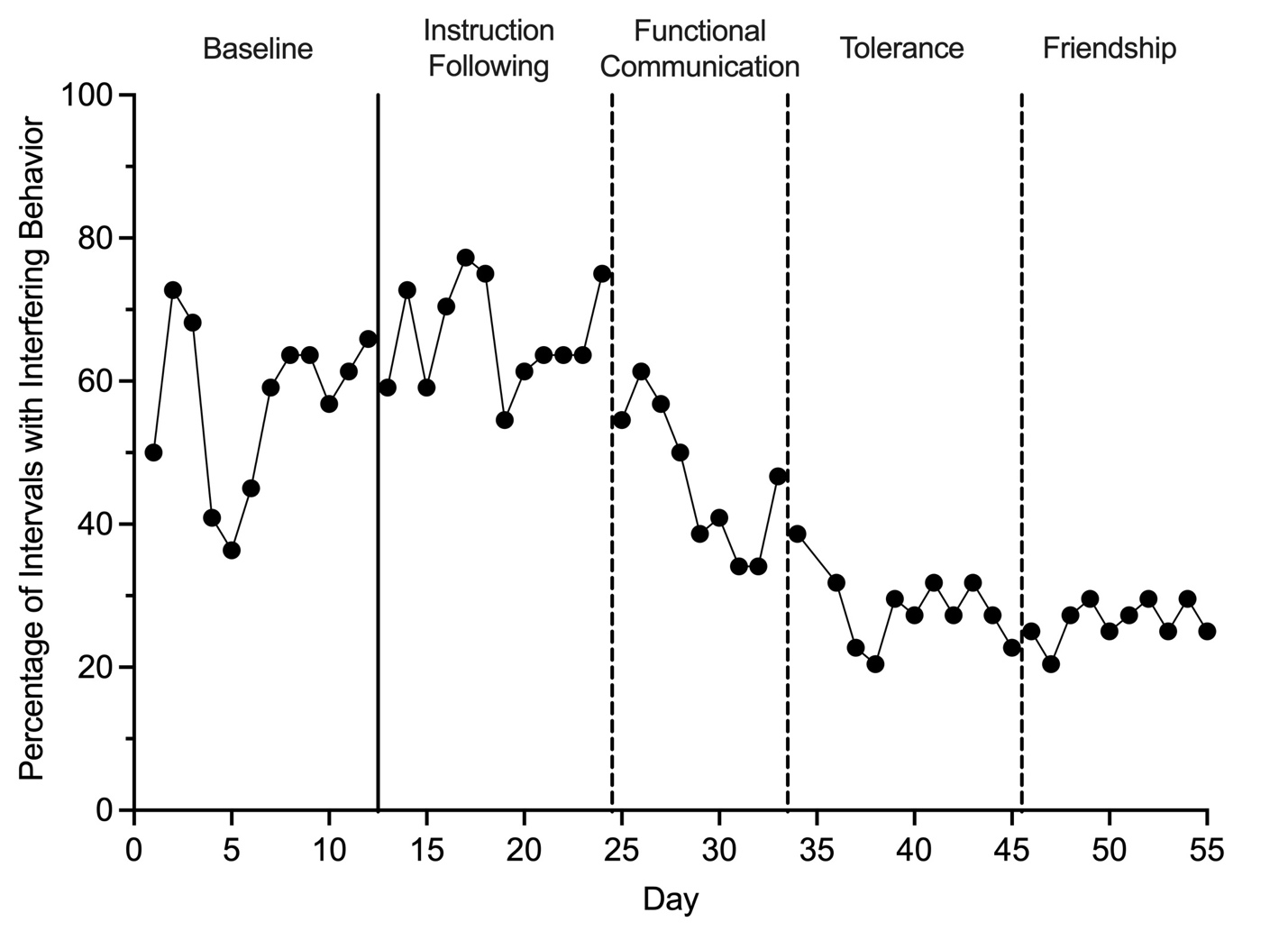

Supplement: Supplementary file 1 — Data S1: Supporting Information [file JABA-59-0-s001.docx]
